# Role of Selenoproteins in Redox Regulation of Signaling and the Antioxidant System: A Review

**DOI:** 10.3390/antiox9050383

**Published:** 2020-05-05

**Authors:** Ying Zhang, Yeon Jin Roh, Seong-Jeong Han, Iha Park, Hae Min Lee, Yong Sik Ok, Byung Cheon Lee, Seung-Rock Lee

**Affiliations:** 1 Department of Biochemistry, Chonnam National University Medical School, Gwangju 501-190, Korea; zhangying4097@163.com (Y.Z.); ip071@hanmail.net (I.P.); 2 Department of Biomedical Sciences, Research Center for Aging and Geriatrics, Research Institute of Medical Sciences, Chonnam National University Medical School, Gwangju 501-190, Korea; 3 College of Life Sciences and Biotechnology, Korea University, Seoul 02841, Korea; ryj0810@korea.ac.kr (Y.J.R.); molhm99@korea.ac.kr (H.M.L.); 4 COTDE Inc. 19-3, Ugakgol-gil, Susin-myeon, Cheonan-si, Chungcheongnam-do 330-882, Korea; hanseongjeong@gmail.com; 5 Korea Biochar Research Center, O-Jeong Eco-Resilience Institute (OJERI) & Division of Environmental Science and Ecological Engineering, Korea University, Seoul 02841, Korea; yongsikok@korea.ac.kr

**Keywords:** selenoprotein, oxidative stress, redox signaling, redox homeostasis, antioxidant

## Abstract

Selenium is a vital trace element present as selenocysteine (Sec) in proteins that are, thus, known as selenoproteins. Humans have 25 selenoproteins, most of which are functionally characterized as oxidoreductases, where the Sec residue plays a catalytic role in redox regulation and antioxidant activity. Glutathione peroxidase plays a pivotal role in scavenging and inactivating hydrogen and lipid peroxides, whereas thioredoxin reductase reduces oxidized thioredoxins as well as non-disulfide substrates, such as lipid hydroperoxides and hydrogen peroxide. Selenoprotein R protects the cell against oxidative damage by reducing methionine-R-sulfoxide back to methionine. Selenoprotein O regulates redox homeostasis with catalytic activity of protein AMPylation. Moreover, endoplasmic reticulum (ER) membrane selenoproteins (SelI, K, N, S, and Sel15) are involved in ER membrane stress regulation. Selenoproteins containing the CXXU motif (SelH, M, T, V, and W) are putative oxidoreductases that participate in various cellular processes depending on redox regulation. Herein, we review the recent studies on the role of selenoproteins in redox regulation and their physiological functions in humans, as well as their role in various diseases.

## 1. Introduction

Most reactive oxygen species (ROS) are generated as by-products of cellular redox processes, including mitochondrial respiration and are known to be harmful to human health when their cellular levels exceed the physiologically acceptable level. However, moderate ROS concentrations play a crucial role in regulating signal transduction and cellular functions, such as proliferation and differentiation, via protein oxidation [1]. Nevertheless, ROS are toxic and can damage various biological molecules, such as proteins, lipids, and nucleic acids. Thus, the imbalance between ROS production and antioxidant capability of the organism is often associated with the development of various chronic pathologies, including cancer, cardiovascular diseases (CVDs), diabetes, neurological disorders, ischemia/reperfusion injury, age-related alterations, dysfunctions related to immune defense and inflammatory responses, and other diseases [1,2,3,4,5,6,7,8,9,10,11].

Antioxidant enzymes such as superoxide dismutase, catalase, and other redox enzymes, including selenoproteins, and low weight antioxidant molecules such as carotenoids, ascorbate, vitamin E, α-lipoic acid, and glutathione (GSH) are essential for maintaining the “steady state” concentration of ROS, which helps to regulate the redox balance and maintain cellular homeostasis. Most functionally characterized selenoproteins have catalytic activities owing to their selenocysteine (Sec) residue and act to neutralize and remove ROS. Therefore, they protect against oxidative stress. Selenium was considered a toxic element for humans and other mammals but is now considered an important trace element, as the benefits of dietary selenium supplementation have been identified [12]. Selenium is widely distributed in various tissues and organs after absorption and performs important biological functions through regulating the synthesis of selenoproteins and being incorporated in selenoproteins [13]. Furthermore, some selenoproteins are also involved in regulating the activation of signaling pathways and cellular functions. In this review, we provide a brief overview of the various functions of selenoproteins and their roles in redox regulation and physiological functions.

## 2. Selenocysteine in Selenoproteins

Sulfur and selenium have similar physicochemical properties as both are members of the chalcogen group and undergo thiol-disulfide exchange reactions in the form of cysteine (Cys) or Sec, respectively [14]. However, Sec is more reactive than Cys under physiological conditions as it has a lower pKa (~5.2) than Cys (~8.0); thus, it can exist as a nucleophile without electrostatic interactions and, therefore, has enhanced catalytic efficiency. The Sec residue in most selenoproteins is located in the catalytic region, where it catalyzes the reduction of oxidized Cys residues, such as disulfide and sulfenic acid [15]. Studies have shown that removal of the Sec residues by oxidative selenium elimination, limited proteolysis [16], as well as specific alkylation of the Sec residues at pH 6.5 [16,17], leads to catalytic activity decrease. Moreover, the substitution of Sec with Cys also results in a marked reduction in catalytic efficiency [18,19,20].

Selenoproteins exist in three kingdoms of life, whereas yeast, fungi, and higher plants lack selenoproteins. Instead, they have alternative cysteine-containing homologs [21]. Sec is the 21st amino acid encoded by the in-frame UGA codon, which is usually recognized as a stop codon; therefore, it requires specialized machinery for its incorporation into proteins. This machinery comprises a selenocysteine tRNA (Sec-tRNA^[Ser]Sec^), a secondary stem-loop structure named selenocysteine insertion sequence (SECIS), SECIS Binding Protein 2 (SBP2), and other protein factors [22,23]. However, its molecular mechanism remains unclear. For Sec-tRNA^[Ser]Sec^ synthesis, selenium can be intaken from dietary sources, including organic forms such as selenomethionine (Se-Met) and inorganic forms such as selenate and selenite [13]. To utilize selenium from Se-Mets, they are converted to Sec by the trans-selenation pathway similar to the trans-sulfuration pathway for Met. Then Sec is converted to H_2_Se by Sec b-lyase [24]. In the case of selenite, it interacts with glutathione and is directly reduced to H_2_Se. Both organic and inorganic selenium sources become H_2_Se and is then converted to selenophosphate, which reacts with tRNA-bound serinyl residues to produce Sec-tRNA^[Ser]Sec^ [25]. In eukaryotes and archaea, SECIS is located in the 3ʹ-untranslated region (UTR) and interacts with *trans*-acting factors [22,26]. This unique feature of SECIS elements and the in-frame UGA codon has been largely adopted for in silico selenoproteome identification in diverse organisms. This is a peculiar feature, considering that another sulfur-containing amino acid Met and Se-Met cannot be distinguished by a Met tRNA, and therefore, Se-Mets are incorporated in proteins randomly [27].

Selenoproteins are essential for survival in many organisms, including humans. For example, prostate epithelium-specific selenocysteine tRNA gene *Trsp* deletion leads to oxidative stress, early-onset intraepithelial neoplasia [28], and early embryonic death in mice [29]. Moreover, mammary gland-specific *Trsp* knockout (KO) mice showed that p53 and BRCA1 expression changed, resulting in enhancing susceptibility to cancer [30], which indicates that selenoproteins are essential for mammals. Based on Sec residue localization, selenoproteins can be divided into two groups. In the first group, which includes all thioredoxin reductases (TrxRs) and selenoprotein I (SelI), SelK, SelO, SelR, and SelS, the Sec residue is located in the C-terminal region. The second group, which contains the rest of the selenoproteins (glutathione peroxidases, iodothyronine deiodinases, SelH, SelM, SelN, SelT, SelV, SelW, SPS2, and Sep15), is characterized by the presence of the Sec residue in the N-terminal region, as part of the redox-active thioredoxin (Trx)-like selenylsulfide/selenolthiol motif [31]. SelP has an N-terminal redox Sec and multiple C-terminal Sec residues [32]. Over half of the mammalian selenoproteins possess the Trx-like fold [33]; its common feature include a two-layer α/β/α sandwich structure and a conserved CXXC motif (two Cys residues separated by two other amino acid residues). The CXXC motif is a “rheostat” in the active site [34], because changes in residues that separate the two cysteines influence redox potentials and p*K*a values of cysteines, configuring proteins for a particular redox function [35]. Altering the CXXC motif affects not only the reduction potential of the protein but also its ability to function as a disulfide isomerase and also affects its interaction with folding protein substrates and reoxidants [20]. The Trx-like fold is commonly observed in proteins, most of which function in disulfide bond formation and isomerization and regulate the redox state of the Cys residues for other functions. Sep15, SelH, SelM, SelO, SelT, SelP, SelW, and SelV contain a CXXU motif, indicating that they have an antioxidant activity, which corresponds to the CXXC motif of the Trx active site. A variety of approaches has been used to determine the biological function of these selenoproteins. However, most selenoproteins (thioredoxin glutathione reductase, SelH, SelI, SelM, SelO, SelT, SelV, SelW) have no known functions. Interestingly, the selenoproteins with identified functions (redox functions) are all oxidoreductases that contain Sec in the catalytic center and participate in various redox processes, such as antioxidant defense, redox signaling, redox regulation of biological functions, and many other processes that regulate intracellular redox homeostasis [31,36,37,38].

## 3. Glutathione Peroxidase

Glutathione peroxidase (GPx) is an intracellular antioxidant enzyme mainly protects the organism against oxidative stress by catalyzing the reduction of hydrogen peroxide, lipid hydroperoxides, and organic hydroperoxides to water or corresponding alcohols, using GSH as an essential cofactor [39]. It has also been reported that GPx dysfunction is associated with the incidence of various types of cancer [40,41], muscle disorders [42], CVDs [43,44], hepatopathies [45], renal failure [46,47], neurological disorders (such as Alzheimer’s disease (AD) and Parkinson’s disease (PD)) [48,49,50], immune defense dysfunction [51], and other diseases.

**Figure 1:**
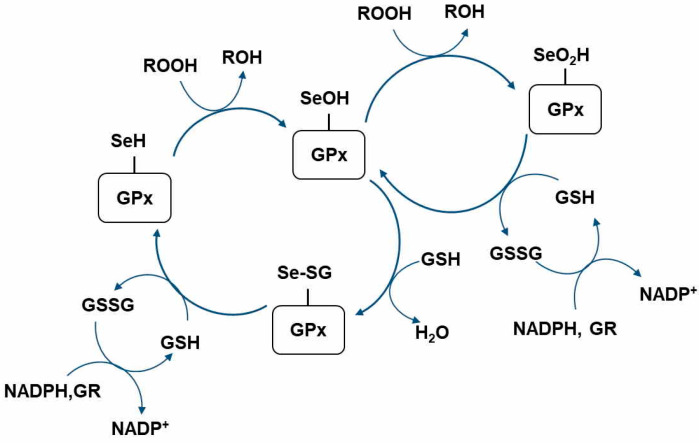
Catalytic redox cycle of glutathione peroxidase. GR, glutathione reductase; GSH, glutathione (reduced form); GSSG, glutathione disulfide; GPx-SeOH, selenenic acid; GPx-SeSG, selenenyl sulfide; GPx-SeO_2_H, seleninic acid; ROOH, hydroperoxides (H_2_O_2_, peroxynitrite or aliphatic hydroperoxide); ROH, H_2_O or corresponding alcohol; NADPH, Nicotinamide adenine dinucleotide phosphate.

The selenol in the Sec residue of GPx is oxidized by H_2_O_2_ or other oxidants, which results in selenenic acid (GPx-SeOH) formation. Then, the GPx-SeOH is converted back to selenol via a two-step process. First, selenenyl sulfide (GPx-SeSG) is produced by the reaction between GPx-SeOH and GSH. Subsequently, the second GSH reduces GPx-SeSG back to selenol. Notably, owing to high levels of oxidative stress or low GSH concentrations, GPx-SeOH may be overoxidized to seleninic acid (GPx-SeO_2_H) (Figure 1).

Mammalian GPx has eight isoforms; of these, GPx1 (ubiquitous, cytosolic), GPx2 (gastrointestinal-specific), GPx3 (plasma), GPx4 (phospholipid hydroperoxide), and GPx6 (olfactory epithelium) contain a Sec residue in the active catalytic site and can, thus, catalyze H_2_O_2_ and lipid hydroperoxide reduction in conjunction with GSH with increased efficiency [52,53]. In contrast, GPx6 homologs in some mammals, GPx5 (epididymal androgen-related protein), GPx7, and GPx8 are not selenoproteins and utilize a conventional Cys residue instead of Sec [54]. GPx1, the first discovered selenoenzyme, is located in the cytosol. Moreover, it is the most abundant GPx and found in nearly all mammalian tissues. GPx1 can reduce H_2_O_2_ and organic hydroperoxides, including *tert*-butyl hydroperoxide and cumene hydroperoxide. Its peroxidase activity and expression are affected by Se status [55]. GPx1 loss is associated with the development of various types of cancer, including breast [41], lung [56], prostate [57], and bladder [58] cancers. GPx2 is an intestinal selenoenzyme highly and weakly expressed in the gastrointestinal mucosal epithelium and human liver, respectively. Moreover, it can catalyze H_2_O_2_, *tert*-butyl hydroperoxide, cumene hydroperoxide, and linoleic acid hydroperoxide reduction [59]. The main function of GPx2 is to protect the intestinal epithelium from oxidative stress and maintain mucosal homeostasis [60]. Florian et al. reported that GPx2 expression levels are much higher in crypt bases than in luminal sites. The crypts contain stem cells that participate in the proliferative zone, thereby suggesting that GPx2 might play a role in cell proliferation. GPx2 loss was also shown to increase apoptosis, mitosis, and GPx1 expression in mice intestines [61]. Gpx3 is the only extracellular secreted member of the GPx family that catalyzes H_2_O_2_, organic hydroperoxides, and lipid hydroperoxides to reduce systemic oxidative stress [18]. GPx3 has been identified as a tumor suppressor in many cancers [62]. GPx3 promoter hypermethylation specifically downregulates its expression, which occurs commonly in human cancers, including prostate, gastric, breast, lung, and colon cancers [63,64,65]. GPx3 dysregulation is also associated with obesity and fat distribution and related to whole body insulin resistance [66]. GPx4 is the only known enzyme that can reduce lipid hydroperoxides, which arise in the membrane. Unlike other GPx, GPx4 not only uses GSH as an electron donor but also uses protein thiols as reducing substrates when GSH becomes limiting [67]. A study has shown that the pro-survival role of selenium in mammals is largely mediated by GPx4 [68]. GPx4 activity is essential to maintain lipid homeostasis, prevent toxic lipid ROS accumulation, and thereby block ferroptosis by its intrinsic resistance to irreversible inactivation [69,70]. GPx6, found in the olfactory epithelium and during embryonic development [54], is highly expressed in the olfactory bulb, striatum, and frontal cerebral cortex [71]. Synthetic lethal screening in the mammalian central nervous system has identified that the age-regulated *Gpx6* gene is a modulator of mutant huntingtin toxicity, and its overexpression can dramatically alleviate both behavioral and molecular phenotypes associated with a mouse model of Huntington’s disease [71].

## 4. Thioredoxin Reductase

**Figure 2:**
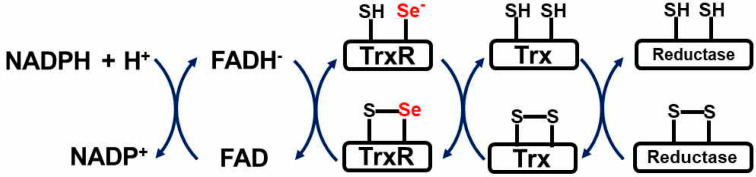
Catalytic redox cycle of the thioredoxin (Trx)/thioredoxin reductase (TrxR)/nicotinamide adenine dinucleotide phosphate (NADPH) system. FAD, flavin adenine dinucleotide.

TrxR is an essential component of the Trx system, which comprises Trx, nicotinamide adenine dinucleotide phosphate (NADPH), and TrxR (Figure 2). The Trx/TrxR system functions as a protein disulfide oxidoreductase to maintain the redox status of intracellular substrate proteins, such as ribonuclease reductase, peroxiredoxin, glucocorticoid receptors, transcription factors, and protein tyrosine phosphatases such as PTEN [72,73]. The two subunits of TrxR are only active in their dimeric form and form a head-to-tail pattern in active homodimeric TrxRs. TrxR is indispensable for Trx function as it is the only enzyme that catalyzes the NADPH-dependent Trx reduction [74]. Three isoforms of TrxR are found in mammals: cytosolic TrxR (TrxR1), mitochondrial TrxR (TrxR2), and a testis-specific thioredoxin glutathione reductase (TrxR3) [75]. All three enzymes contain conserved Gly-Cys-Sec-Gly sites in the flexible C-terminal region, which is both reactive and solvent-accessible [19,76,77,78,79,80]. In particular, the Sec residue is critical for TrxR reductase activity [81]. Mammalian TrxR1 and TrxR2 have another conserved site, namely the Cys-Val-Asn-Val-Gly-Cys motif, which is adjacent to the flavin adenine dinucleotide located in the N-terminal region [19,77]. Owing to these two conserved sites, mammalian TrxRs possess surprisingly multifaceted properties and functions beyond direct Trx reduction. In addition to Trx, mammalian TrxRs have a broad substrate specificity. As such, they can reduce glutaredoxin 2, protein disulfide isomerase, and many other disulfides in proteins. They also reduce some non-disulfide substrates, such as H_2_O_2_ [21], selenite [82], lipid hydroperoxides [83], ascorbic acid [84], α-lipoic acid [74,85,86,87], cytosolic peptide granulysin [88], antibacterial NK-lysine [89], dehydroascorbate [84], and cytochrome C [90]. Similar to other selenoproteins, such as SelR and GPx1, the expression of TrxRs depends on the concentration of selenium: selenium deficiency reduced TrxR synthesis; however, a high selenium concentration mediated Sec incorporation and increased TrxR enzyme activity without increasing protein synthesis [91,92].

TrxRs are ubiquitously expressed enzymes that regulate redox metabolism and play a critical role in protection against malignant transformation. There is increasing evidence that supports the idea that TrxRs inhibit multiple stages of tumor progression, from initiation to growth, invasion, and metastasis [93,94,95]. Interestingly, TxR overactivation or dysfunction is associated with the onset of various diseases, such as CVDs, neurological disorders, type 2 diabetes, human immunodeficiency virus infection, and cancer [96,97,98,99]. Trx1 not only acts as an antioxidant but also plays an important role in cellular function by regulating signaling pathways via direct interaction with other small molecules, all of which are involved in ventricular remodeling inhibition after myocardial infarction [100]. Therefore, in CVD, TrxR functions via interactions with Trx1 [101].

**Figure 3:**
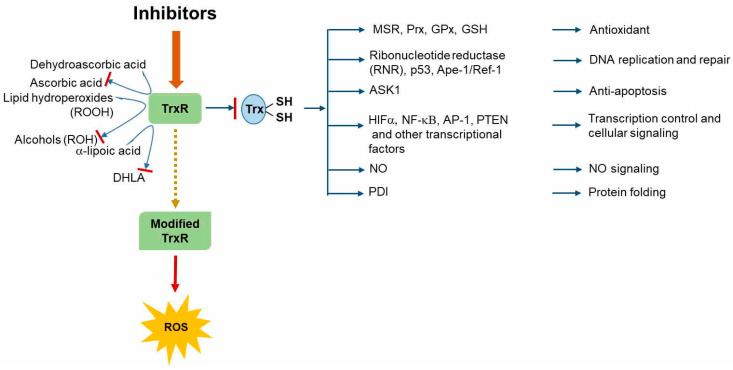
Thioredoxin reductase (TrxR) as a novel target for cancer therapy. Gold compounds, nitrosoureas, arsenic trioxide, dinitrohalobenzenes, curcumin, flavonoids, quinones, and other cancer chemotherapeutics have been shown to be TrxR inhibitors. TrxR inhibition blocks thioredoxin (Trx)-mediated activity in various processes, such as defense against oxidative stress, DNA replication and repair, apoptosis inhibition, transcription control, and protein folding via different signaling pathways. Some inhibitors, such as 1-chloro-2,4-dinitrobenzene (DNCB) and curcumin, modify TrxR via the alkylation of Cys^496^ and redox-active Sec^497^ residues and induce nicotinamide adenine dinucleotide phosphate (NADPH) oxidase activity, finally leading to reactive oxygen species (ROS) production.

The tumor suppressor PTEN negatively regulates the PI3K/AKT signaling pathway, which is pivotal for cell growth and survival. Numerous studies have demonstrated that PTEN catalytic activity is regulated via direct oxidation by ROS [102,103]. Therefore, its enzymatic activity recovery depends on cellular Trx/TrxR system availability [73]. Increased Trx1 expression in human tumors is associated with abnormal growth, which is caused by the binding of Trx1 to the C2 domain of PTEN, thereby resulting in the inhibition of its lipid phosphatase activity and membrane binding capacity [104]. It has been reported that Trx and TrxR are highly overexpressed in a variety of aggressive tumors and may increase tumor cell survival and proliferation [105,106,107], indicating that the Trx system has a dual function in cancer. The effect of TrxR inhibition on tumor cell survival and aggressiveness is robust, and tumor proliferation appears to be dependent on an active Trx system, making TrxR a potential target for cancer chemotherapy [108,109,110,111,112] (Figure 3). In this regard, the Sec residue in mammalian TrxR can be the primary target for the development of drugs that exert inhibitory effects on various type of cancers, including gold compounds and platinum-based drugs [113], alkylating anticancer agents such as nitrosoureas [114], nitrogen mustards, ifosfamide [115], and cyclophosphamide [116], arsenic trioxide [117], dinitrohalobenzenes such as 1-chloro-2,4-dinitrobenzene, 1-fluoro-2,4-dinitrobenzene, and 1-bromo-2,4-dinitrobenzene, and natural products such as curcumin [118], flavonoids [119], and quinones [120]. The irreversible TrxR inhibition by dinitrohalobenzenes and curcumin is accompanied by the alkylation of both the redox-active Sec^497^ and its neighboring cysteine residue Cys^496^. Moreover, this modified TrxR strongly induces NADPH oxidase activity, which leads to ROS production [118,121].

## 5. Selenoprotein R

SelR (also designated as MsrB1) is an antioxidant enzyme that uses Met to defend cellular macromolecules against oxidative stress. Met is a sulfur-containing amino acid that is readily oxidized to Met sulfoxide by ROS; subsequently, Met sulfoxide reductases (Msr) such as SelR reduce Met sulfoxide back to Met [122]. Met sulfoxide contains two diastereomeric forms, Met-S-sulfoxide (Met-S-SO) and Met-R-sulfoxide (Met-R-SO) [123]. Met-R-SO is reduced by the MsrB family of proteins, including SelR, whereas Met-S-SO is reduced by the MsrA family of proteins [124]. Mammals have one MsrA and three MsrBs, namely, SelR, MsrB2, and MsrB3 [18]. Among these, SelR is the only selenoprotein that is localized in both the cytosol and nucleus. SelR is present specifically in vertebrates and appears to have evolved separately, having the lowest homology with other Msr enzymes [125].

**Figure 4:**
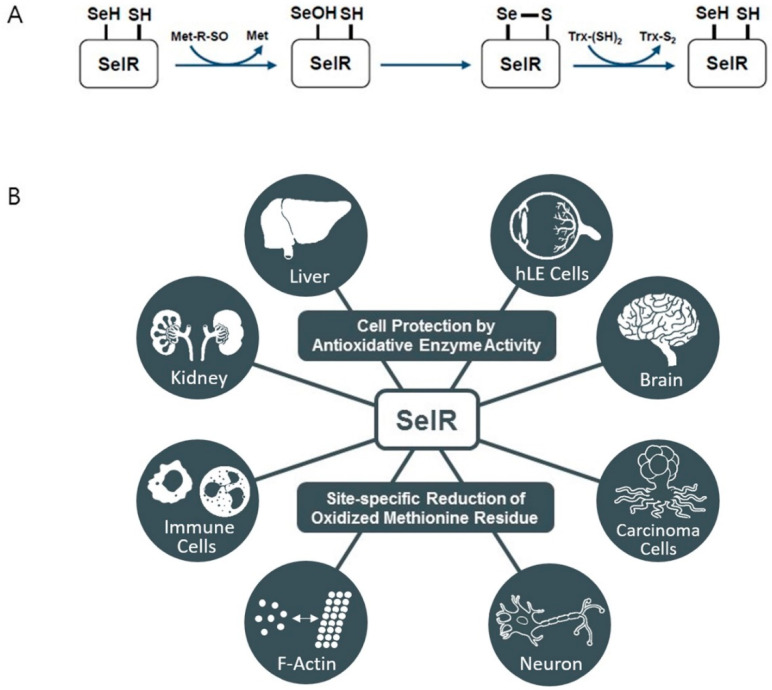
(**A**) Catalytic mechanism of SelR reducing methionine-R-sulfoxide (Met-R-SO). The catalytic selenocysteine (Sec) residue attacks Met-R-SO and forms the intermediate selenenic acid with Met release. The resolving cysteine (Cys) residue attacks the intermediate, resulting in the formation of intramolecular selenide–sulfide bond. The intramolecular selenide–sulfide bond of SelR is directly reduced by thioredoxin (Trx) system. (**B**) Role of SelR in various organs and cell types.

SelR expression is regulated by dietary selenium; its mRNA expression level is low in a selenium-deficient diet, but this can be reversed by dietary selenium supplementation [126]. SelR activity was also found to reduce with age [127]. SelR has catalytic activity, especially for protein-bound and free Met-R-SO but has low catalytic efficiency. Like other Msr enzymes, SelR is an oxidoreductase that requires Trx/TrxR/NADPH to recycle its oxidized form to the reduced form (Figure 4A) [99]. Along with its catalytic activity toward protein-bound Met-R-SO, SelR plays a role in repairing oxidized proteins, thus protecting the structure and function of proteins against oxidative stress [128]. SelR also regulates biological processes via the reversible oxidation/reduction of Met residues in proteins. The oxidation of Met residues at certain sites by either ROS or enzymes often leads to changes in protein function, which can then be reversed by SelR-catalyzed reduction of the said Met residues [129]. For instance, it was found that F-actin disassembly caused by the stereospecific oxidation of the 44 and 47 Met residues in actin by MICAL proteins can be rescued by SelR [130]. Actin cytoskeleton dynamics regulation is important for many cellular responses, including neural development, muscle contraction, and filopodia formation [131,132,133]. Moreover, F-actin assembly is known to be bidirectionally associated with the mitogen-activated protein kinase (MAPK) pathway, which controls many cellular processes, including cell proliferation [134]. Accordingly, SelR is a potentially redox-dependent regulator that participates in many cellular processes and signaling pathways related to actin cytoskeleton dynamics via F-actin assembly regulation.

SelR KO mice exhibit increased oxidative stress in the liver and kidney with exacerbated hepatotoxicity [135,136]. SelR is also required for human lens epithelial (hLE) cell viability against oxidative stress-induced apoptosis and attenuates cataracts [137]. Since membrane-bound proteins in hLE cells from patients with cataract contain high levels of Met sulfoxide residues, SelR may directly retard cataract [138]. SelR appears to play an important role in innate immunity; however, its underlying mechanism is poorly understood. In macrophages, SelR expression is induced by lipopolysaccharides and is involved in controlling macrophage function by promoting the expression of anti-inflammatory cytokines, such as IL-10 and IL-1RA [139]. Neutrophils were also shown to have high levels of SelR expression in response to excessive ROS. Moreover, a recent study has suggested that decreased SelR activity in neutrophils might be associated with AD [140]. A study has also shown that SelR is highly expressed in carcinoma cells in response to increased oxidative stress, and may thus enhance carcinoma cell survival. Moreover, SelR expression upregulation aggravates oncogenesis by promoting proliferation via MAPK pathway activation and promotes invasion and metastasis by regulating actin cytoskeleton dynamics [141,142] (Figure 4B).

## 6. Selenoprotein O

**Figure 5:**
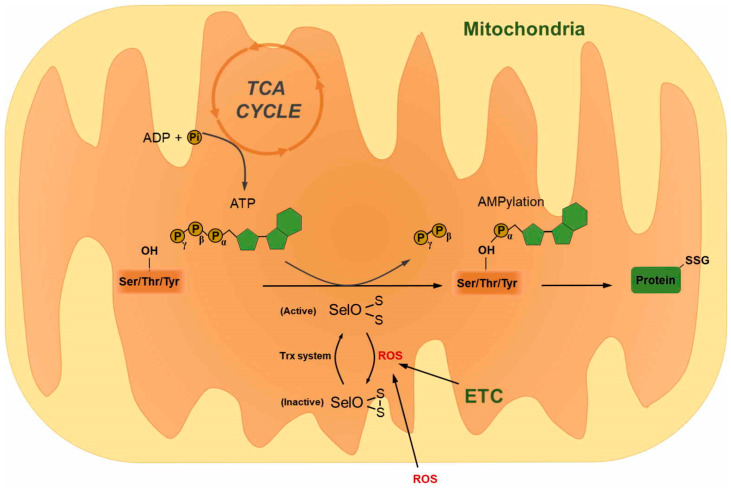
Selenoprotein O (SelO) mediates protein AMPylation and protects the cell from oxidative stress.

SelO, the largest protein among the 25 mammalian selenoproteins, is expressed in a variety of organs, such as the brain, heart, liver, kidneys, lungs, and stomach [54,143]. Unlike SelR and GPx1 expression, SelO expression is not influenced by a selenium-deficient diet [143]. In higher eukaryotes, SelO contains a single Sec residue near the C-terminal region [54,143]. Notably, in lower eukaryotes and all prokaryotes, the Sec residue in SelO is replaced with an invariant Cys residue [144]. Mammalian SelO is located in the mitochondria [143,144], and the occurrence of the CXXU motif in the C-terminal region suggests that SelO might have a redox-active Sec residue, similar to other thiol-dependent oxidoreductases [143]. SelO activity in *Escherichia coli* is regulated by intramolecular disulfide bridge formation between a Cys residue in the activation loop (Cys272) and the Cys residue in the C-terminal region (Cys476), with the latter being replaced by a Sec residue in higher eukaryotes [144]. Using bioinformatic tools, Dudkiewicz et al. predicted that the three-dimensional structure of SelO may be similar to that of a protein kinase and that it might have phosphotransferase activity [145]. Recently, structural studies have shown that SelO is a highly conserved pseudokinase that transfers AMP from ATP to Ser, Thr, and Tyr residues in its substrate protein via a process known as AMPylation [144]. SelO plays a role in response to oxidative stress and regulates global S-glutathionylation levels via AMPylation in conjunction with glutaredoxin [144] (Figure 5). Furthermore, SelO has been shown to play an essential role in chondrocyte viability, proliferation, and chondrogenic differentiation [146]. However, the physiological functions of SelO remain unknown. As such, further research is needed to clarify its physiological functions, role in disease, and association with other redox enzymes.

## 7. Other Selenoproteins

SelS (also designated as SEPS1, VIMP, and Tanis) is a single-pass transmembrane protein [54] that has an extensive tissue distribution, being present in the liver, kidneys, adipose tissue, skeletal muscle, pancreatic islets, and blood vessels [147]. SelS participates in the endoplasmic reticulum (ER)-associated protein degradation (ERAD) pathway, which is responsible for transporting unfolded or misfolded proteins from the ER to the cytoplasm, followed by degradation via the ubiquitin–proteasome system [148]. SelS is a Trx-dependent reductase that catalyzes H_2_O_2_ and cumene hydroperoxide reduction [149]. An NF-κB-binding site is located within the SelS gene promoter region in Bama mini-pigs [150]. Moreover, SelS can regulate the production of inflammatory cytokines, such as IL-1β and IL-6, in stimulated astrocytes [151]. This suggests that SelS is involved in inflammation, oxidative stress, and endoplasmic stress [152,153].

Human SelT is a 22 kDa protein localized to the Golgi apparatus and ER and present in the plasma membrane [154]. SelT possesses a Trx-like fold and a conserved CXXU motif, which are common structural domains in oxidoreductases with a catalytic Sec residue. Moreover, SelT knockdown was found to increase the expression of Cbr3 and SelW, which are involved in redox regulation, thereby supporting the idea that this protein might function as an oxidoreductase [155]. SelT is a trophic neuropeptide pituitary adenylate cyclase-activating polypeptide (PACAP)-regulated gene involved in intracellular Ca^2+^ mobilization and neuroendocrine secretion. Sec-containing SelT overexpression in PC12 cells was found to increase intracellular Ca^2+^ concentrations, whereas the Sec-to-Ala SelT mutant overexpression had no effect on Ca^2+^ release, suggesting that SelT regulates intracellular Ca^2+^ mobilization via the redox-active Sec residue [156]. SelT has also been reported to protect dopaminergic neurons against oxidative stress and prevent early and severe movement impairment in Parkinson’s disease (PD) animal models [157].

SelN is a 65 kDa transmembrane glycoprotein, which is localized to the ER that contains a transmembrane-addressing site in proximity to the EF-hand motif, which is a helix-loop-helix structural motif found in a large family of calcium-binding proteins. [158]. Human SelN mRNA is detected in most fetal tissues, but its level reduces in adult tissues [159]. Moreover, its expression increases in proliferating cells, such as fibroblasts and myoblasts, and gradually decreases during the differentiation of myoblasts to myotubes [158]. SelN is so far the only selenoprotein directly linked to human genetic disorders. Certain mutations in the SelN gene cause SEPN1-related myopathy (SEPN1-RM), which is an early-onset muscle disease that is characterized by muscle weakness, spinal rigidity, and respiratory insufficiency. SelN plays an important role in conferring resistance against oxidative stress and maintaining Ca^2+^ homeostasis in human skeletal muscle cells [160]. Moreover, SelN-deficient fibroblasts and muscle cells have been shown to have an increased susceptibility to H_2_O_2_-induced oxidative stress [160]. Notably, in SelN-deficient muscle cells, the generated ROS/NO have been found to regulate intracellular Ca^2+^ concentrations via the modulation of Ca^2+^ channels, followed by Ca^2+^ release or leaking [160].

## 8. Conclusions

Organisms contain an array of defense systems, such as the thiol-dependent antioxidant system, which coordinate to remove ROS and reactive nitrogen species. This review focused on several mammalian selenoproteins, discussing their splicing forms, structures, and relationships with oxidative stress and disease. Although the functions of some selenoproteins still remain unclear, up-to-date research is advancing in the characterization of some of the less known selenoproteins, as well as their role in the development of various diseases as they may act as potential drug targets. Further studies should focus on revealing the detailed molecular mechanisms underlying the functions of selenoproteins, which can further help develop new guidelines for novel therapies in various diseases.
